# Experimental study on the effect of room temperature pre-oxidized time on spontaneous combustion characteristics of coal

**DOI:** 10.1038/s41598-023-48301-3

**Published:** 2023-12-12

**Authors:** Xun Zhang, Huimin Liang, Ge Huang, Bing Lu, Chen Yu, Jiahui Zou

**Affiliations:** 1 College of Mining, Liaoning Technical University, Fuxin, 123000 Liaoning China; 2 College of Safety Science & Engineering, Liaoning Technical University, Fuxin, 123000 Liaoning China

**Keywords:** Coal, Energy, Infrared spectroscopy

## Abstract

The presence of different types of coal at room temperature can lead to self-heating of coal, potentially resulting in spontaneous combustion. To investigate the effect of ambient temperature pre-oxidation (BL) time on the self-combustion characteristics of different coal types, synchronous thermal analysis (STA) and Fourier-transform infrared spectroscopy (FTIR) experiments were conducted. The results of the synchronous thermal analysis experiments indicate that ambient temperature pre-oxidation for 3 months (BL3), BL6, and BL9 coals exhibit faster oxidation reactions compared to the original coal, while BL12 coal shows slower oxidation than the original coal. Among these, BL9 coal demonstrates the most significant changes in oxidation reaction characteristics, with the fastest oxidation reaction time being 35.36 min, which is 1.38 min faster than the original coal. To support this observation, a comparison was made between the relative content of active functional groups in the original coal and BL coal. The study revealed that the BL process affects the relative content of hydroxyl groups, aromatic hydrocarbons, aliphatic hydrocarbons, and oxygen-containing functional groups, thereby influencing the coal-oxygen reaction process. This suggests that pre-oxidized coal, compared to the original coal, has a larger pore structure, which plays a dominant role in promoting coal self-combustion in the first 9 months of the BL process. As BL time continues to increase, the continuous reaction of active functional groups at room temperature leads to excessive consumption, resulting in a more significant role in inhibiting coal self-combustion. The research results provide valuable insights for predicting the spontaneous combustion risk of oxidized coal.

## Introduction

The mine disasters caused by spontaneous coal combustion result in significant economic losses and casualties^1,2^. In China, more than 50% of coal mines mining coal seams are at risk of spontaneous combustion and fires^3^, and in mine fire incidents, over 90% are attributed to coal self-ignition^4^. Therefore, investigating the patterns of coal spontaneous combustion is essential to provide theoretical guidance and a scientific basis for the prevention and control of mine fires^5^.

During mining, storage, and transportation, coal is easily oxidized when it comes into contact with oxygen. The presence of pre-oxidized coal and its propensity for spontaneous combustion constitutes a danger to the safety of coal mining. For instance, explosions are prone to occur during the re-opening of sealed fire areas, frequent coal fires occur in goaf areas, and extinguished coalfield fires may rekindle after a certain period. These phenomena indicate an increased risk of self-ignition associated with pre-oxidized coal. To better mitigate the hazards of spontaneous combustion of pre-oxidized coal, many scholars at home and abroad have studied the effects of oxygen concentration and temperature on spontaneous combustion characteristics of pre-oxidized coal respectively.

In terms of oxygen concentration, Zhong et al.^6–8^ analyzed the low-temperature oxidation kinetic properties of pre-oxidized coal at various concentrations of oxygen using TG-DSC and found that the differences in kinetic parameters at different oxygen concentrations were very small. Qi et al.^9^ used thermodynamic methods to analyze the oxidizing characteristics of pre-oxidized coal under a low-oxygen atmosphere in a targeted manner. They discovered that oxygen concentration has varying effects on combustion characteristics and kinetic parameters during different oxidation stages. Wang et al.^10,11^ carried out programmed temperature tests to investigate the effect of different concentrations of oxygen on coal-release gas production. The findings indicated that gas products produced by coal decreased significantly as the oxygen concentration increased. Zhou et al.^12^ further analyzed the effect of oxygen concentration upon the reactive functional groups in pre-oxidized coal by using a self-developed simulation laboratory station for low-temperature oxidation of coal. They found that the hydroxyl group in coal changed most significantly at different oxygen concentrations.

As for the impact of temperature on pre-oxidized coal, Wang et al.^13^ investigated the variation of oxidation characteristics of pre-oxidized coal in the heating as well as in the cooling through programmed temperature experiments and found that the variation in the cooling process was more significant, which was mainly manifested in the dramatic fluctuation of oxygen consumption, and the lower the oxygen concentration, the higher the oxygen consumption. Niu et al.^14^ used TG-DSC to analyze self-ignition characteristics during secondary oxidation in coal at different pretreatment temperatures using techniques such as thermogravimetric analysis and differential scanning calorimetry (TG-DSC). The results of the study show that both the surface and accelerated oxidation phases of coal can have a significant impact on its spontaneous combustion characteristics. Guo et al.^15,16^ analyzed the changes in thermal characteristic parameters and active functional groups of coal at different pre-oxidation temperatures by programmed temperature experiments and FTIR. The findings indicated that the heat diffusivity of coal decreased at different peroxidation temperatures, while the specific heat volume and specific thermal conductance first increased and subsequently decreased. In addition, peroxidation promoted the decomposition of aromatic hydrocarbons in coal. Lv et al.^17^ investigated the characteristic temperature variations in coals at different pre-oxidation temps by TG-DSC and they found that pre-oxidized coals exhibit a critical oxidation temperature; above this temperature, the risk of spontaneous combustion increases and vice versa.

The mentioned research primarily focuses on the impact of pre-oxidation temperature and oxygen concentration on the gaseous products of secondary oxidation and the microstructure of pre-oxidized coal. However, coal exhibits a complex and diverse molecular structure, with various functional groups in coal engaging in competitive and parallel reactions. The long-term BL times are expected to have a certain influence on coal-oxygen complex reactions.

Therefore, it is essential to investigate how BL time affects the thermal effects and microstructure of pre-oxidized coal to comprehensively understand the multiscale behavioral characteristics of coal spontaneous combustion. In this study, pre-oxidized coal samples with varying degrees of metamorphism were prepared and subjected to STA experiments and FTIR experiments. The analysis examined the thermal weight loss characteristics, thermal effects, and changes in the chemical structure of coal under different BL times, aiming to elucidate the mechanisms through which BL time influences coal spontaneous combustion. This study provides a useful reference for predicting the spontaneous combustion hazard of oxidized coal.

## Experiments and methods

### Coal sample

Coal samples were collected from three different coal types: Pingzhuang lignite (abbreviated as HM), Lijiahao long-flame coal (abbreviated as CYM), and Sitaicoking coal (abbreviated as JM), which represent varying degrees of metamorphism. The on-site collected coal samples were immediately sealed in bags and transported to the laboratory for further processing. In the laboratory, the freshly mined coal from the above-mentioned mines was crushed, and coal particles with sizes ranging from 150 to 200 mesh were sieved for use in the experiments. Subsequently, 20 g of each coal sample was placed in glass dishes and subjected to pre-oxidation at room temperature for 3, 6, 9, and 12 months, respectively, to serve as experimental coal samples. Additionally, 20 g of the original coal was placed in sealed glass bottles and stored in the same environment as control group samples. Before the experiments, all coal samples were subjected to vacuum drying at 40 °C for 48 h to remove external moisture, ensuring consistency in moisture content between the experimental coal samples and the original coal and eliminating the influence of moisture on the experimental results. Finally, the prepared coal samples were stored in sealed bags and vacuum-packed for future testing. Industrial analysis and elemental analysis of different coal samples are detailed in Table 1. For convenience in the subsequent discussion, the coal samples subjected to room-temperature pre-oxidation for 3, 6, 9, and 12 months are denoted as BL3, BL6, BL9, and BL12, respectively.

**Table 1:** Industrial and elemental analysis of coal.

Coal sample	Proximate analysis (mass%)				Ultimate analysis (mass%)			
	M_ad_	A_ad_	V_ad_	FC_ad_	C_daf_	H_daf_	O_daf_	N_daf_
HM	9.85	11.35	33.69	45.11	58.08	5.85	34.56	1.51
CYM	7.22	11.71	30.56	50.51	73.62	4.81	20.56	1.01
JM	2.69	5.28	20.74	71.29	82.69	3.94	12.76	0.61

### STA experiment

**Figure 1:**
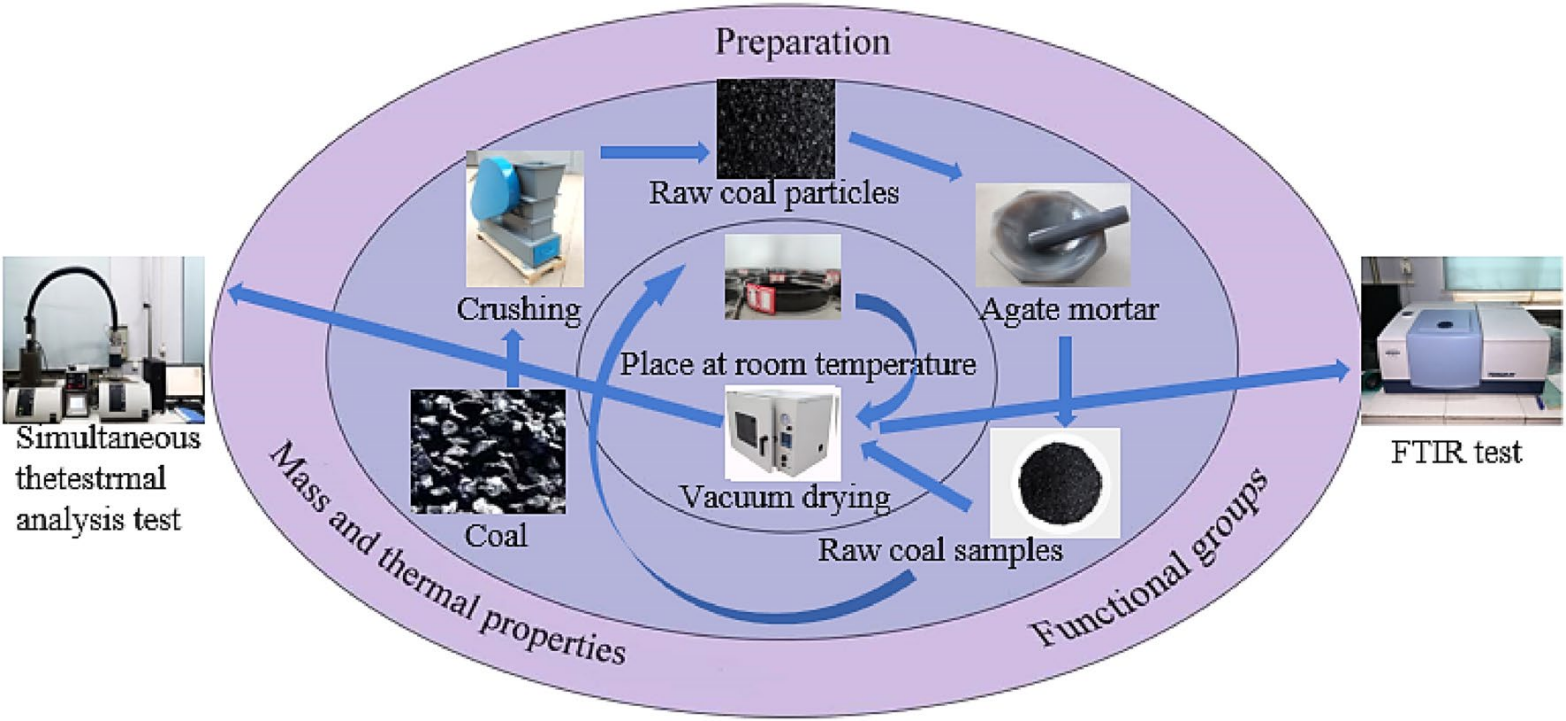
Experimental methodology overview.

As shown in Fig. 1, the experiment employed a non-isothermal thermogravimetric method. The reaction gas used was oxygen with a flow rate of 10 ml/min, while the protective gas was nitrogen with a flow rate of 40 ml/min. The heating rate was set at 5 °C/min, and the reaction temperature ranged from 30 °C to 800 °C. The samples were placed in aluminum oxide crucibles with a mass of 10 mg. The experimental instrument utilized the STA449C comprehensive thermogravimetric analyzer.

### FTIR experiment

As shown in Fig. 1, the experiment utilized the German TENSOR27 FTIR spectrometer. The potassium bromide was dried in an oven at 110 °C for 4 h and then the coal samples were ground in a mortar and pestle at a ratio of 1:180 with the potassium bromide. The mixture is thoroughly ground and then pressed using a tablet press. Tableting was carried out to obtain transparent and homogeneous tablet samples. Finally, the prepared flake samples were tested in ambient air using FTIR spectroscopy. The spectral range for this experiment was set from 400 to 4000 cm^−1^ with a definition of 4 cm^−1^. In total, 32 scans have been performed to obtain FTIR data.

### Analysis method

#### Definition and division of characteristic temperature and characteristic stage in coal

The TG and DSC curves are derived from the thermal analysis experiments, and the first-order derivatives of the TG curves yield the DTG curves, which are utilized to characterize an apparent rate of change in the apparent mass of the coal. The DSC curve's integral area provides a quantitative measure of the heat absorption or heat release demonstrated by the coal sample. Furthermore, by performing a first-order derivative on the DSC curve, the DDSC curve provides a deeper understanding of the variations in the heat release or heat absorption rates observed in coal^18–21^.

**Figure 2:**
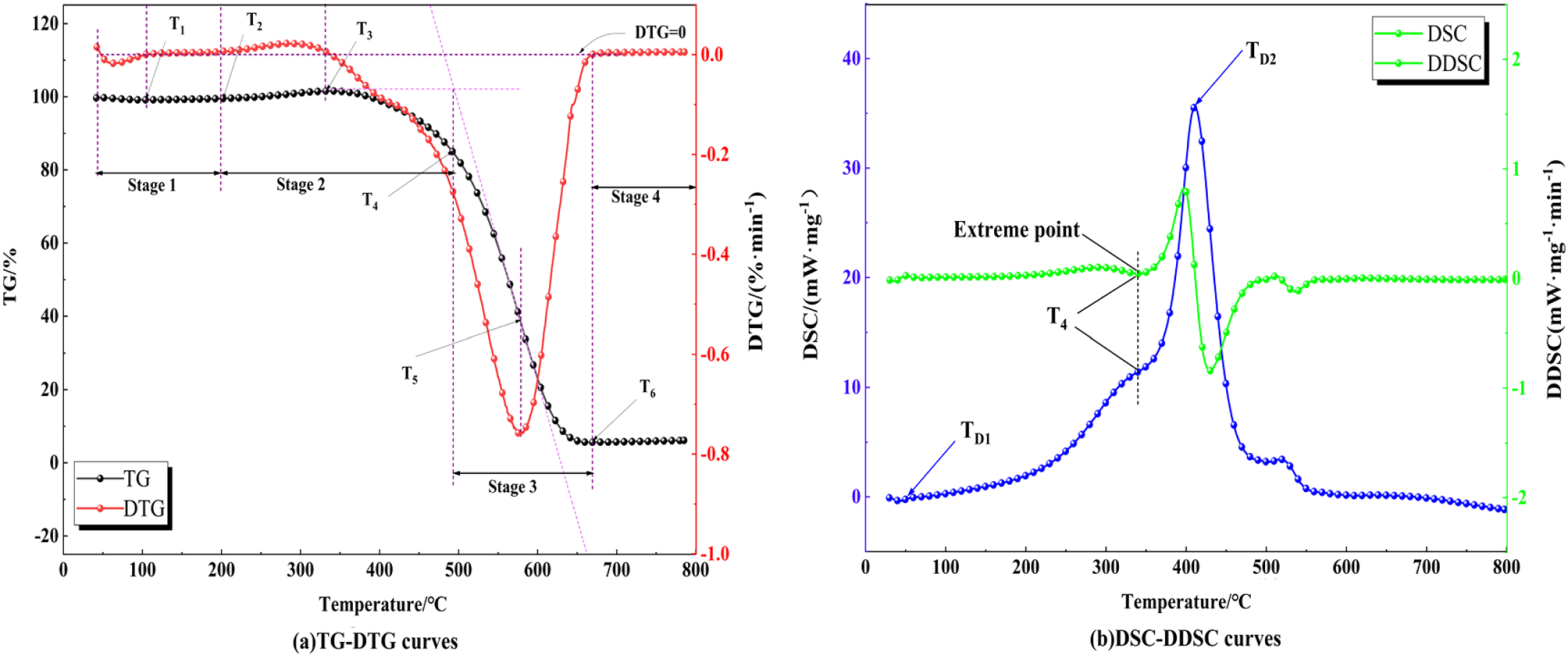
The division of characteristic temperature and characteristic stage in coal.

After inspecting the TG-DTG and DSC-DDSC curves, the selected characteristic temperatures and stages are shown in Fig. 2a and b. T_1_ represents the dry cracking temperature, which corresponds to the temperature at which the coal exhibits its minimum mass on the TG curve before ignition. It marks the completion of water transpiration and the start of oxygen uptake as well as the stage of mass gain. T_2_ denotes the active temperature, at which the coal transitions from a state of single physical adsorption with oxygen to a coexistence of physical and chemical adsorption. The point at T_3_ (peak mass temperature) corresponds to the point on the TG profile where mass attains its highest value during coal oxidation. The firing temperature (T_4_) represents the most significant change in heat emission rate on the DSC profile^22^. At T_4_, the sample started to combust. The temperature that corresponds to the lowest spot of the DTG curve is called T_5_ (temperature of maximum weight loss rate). At T_6_, the temperature at the end of the combustion weight loss peak on the DTG curve is considered the burnout temperature.

In the DSC curve, T_D1_ represents the initial hot spot, and the value of the DSC curve is greater than 0. T_D2_ represents the temperature with the highest rate of heat dissipation. Therefore, in the TG-DTG curve, the oxidation and combination are classified into four stages according to the characteristic temperatures: the first stage involves the water vaporization as well as the desorption of gases (from 30 °C to T_2_), the second stage is the process of slowly oxidizing reaction (from T_2_ to T_4_), the third stage is the process of burning (from T_4_ to T_6_), and the fourth stage is the process of combustion exhaustion (from T_6_ to 800 °C). In the DSC-DDSC curve, the low-temperature oxidation in coal is classified into two characteristic stages: the accelerated oxidation stage (T_D1_ to T_4_) and the rapid oxidation stage (T_4_ to T_D2_).

#### Definition of combustion characteristic index in coal

To provide a more accurate description of the heat changes during the low-temperature oxidation stage of coal, we introduced certain parameters and conducted computational analysis.

Among these indices, the maximum rate of weight loss (DTGmax) assumes a crucial role in reflecting the quality of coal. This parameter can be expressed using Eq. (1):

1 $$DTG_{{\max }} = dW_{{\max }} = \left( {\frac{{dw}}{{dt}}} \right)_{{\max }} .$$

In the formula, dw/dt represents the changing rates of change of quality-led loss (%/min).

The D is employed to characterize the reactivity and burn-up capacity of coal during the later stages of oxidative combustion. This parameter can be represented using Eq. (2) as follows^23,24^:

2 $${\text{D = 10}}^{{5}} \frac{{\left( { - \frac{{{\text{dw}}}}{{{\text{dt}}}}} \right)_{{{\text{max}}}} }}{{{\text{T}}_{{4}} {\text{T}}_{{5}} \frac{{\Delta {\text{T}}_{{6}} }}{{\Delta {\text{T}}_{{1/2}} }}}},$$

where: ΔT_1/2_ represents the half-peak width of the weight loss rate peak of the coal combustion DTG curve, °C; ΔT_6_ represents the temperature interval corresponding to the temperature range where dw/dt reaches its maximum value dw/dt_max_, and the derivative becomes half of the maximum value ${({\text{dw}}/{\text{dt}})/({\text{dw}}/{\text{dt}})}_{\text{max}}\text{=0.5}$, after the maximum weight loss peak of the DTG combustion curve.

In the STA experiments for coal, following the determination of various characteristic temperature points, three comprehensive combustion performance parameters, namely C_b_, S, and H_f_, were introduced to better characterize the coal's combustion characteristics. C_b_ represents the coal's reactivity in the early stages of oxidation and combustion, while S characterizes the combustion characteristics throughout the entire oxidation process. H_f_, on the other hand, signifies the stability of coal combustion. These parameters are expressed by Eqs. (3), (4), and (5)^25^:

3 $${\text{C}}_{\text{b}}\text{=}{10}^{5}\frac{{\left(-\frac{\text{dw}}{{\text{dt}}}\right)}_{\text{max}}}{{\text{T}}_{4}^{2}}$$

4 $$\text{S} = {10}^{7}\frac{{\left(\frac{\text{dw}}{{\text{dt}}}\right)}_{\text{max}}{\left(\frac{\text{dw}}{{\text{dt}}}\right)}_{{\text{mean}} \, }}{{{\text{T}}_{4}}^{2}\cdot {\text{T}}_{6}}$$

5 $${\text{H}}_{{\text{f}}} { = }\frac{{{\text{T}}_{{{\text{max}}}} }}{{{1000}}}{\text{ln}}\left( {\frac{{\Delta {\text{T}}_{{1/2}} }}{{\left( {\frac{{{\text{dw}}}}{{{\text{dt}}}}} \right)_{{{\text{max}}}} \left( {\frac{{{\text{dw}}}}{{{\text{dt}}}}} \right)_{{{\text{mean}}}} }}} \right),$$

where: T_max_ is the experimental maximum temperature, °C; (dw/dt)_max_ and (dw/dt)_mean_ are the maximum and average weight loss rates, %/min, respectively.

#### Kinetic analysis

The spontaneous combustion of coal is a typical gas–solid heterogeneous dynamic reaction process^26,27^. Ballester et al.^28^ conducted relevant research, utilizing a kinetic model to calculate the dynamic parameters of different stages in the coal oxidation process by analyzing the transformation rate characterized by mass loss and heat release. In coal oxidation studies, there are various methods available to determine the kinetics of non-isothermal decomposition^29^. However, in this study, dynamic parameters were determined using non-isothermal DSC (Differential Scanning Calorimetry) curves obtained at a heating rate of 10 °C/min. The kinetic equation can be expressed as Refs.^30,31^:

6 $${\text{d}}\alpha {\text{/dT = }}\left( {{1/}\beta } \right){\text{Aexp}}\left( { - {\text{E/RT}}} \right)\left( {{1} - \alpha } \right)^{{\text{n}}}$$

7 $$\alpha \text{=}\frac{{\text{m}}_{0}-{\text{m}}_{\text{b}}}{{\text{m}}_{0}},$$

where α represents the conversion rate; T stands for coal temperature in Kelvin (K); β denotes the heating rate, with a specific value of β = 10 °C/min; A is the pre-exponential factor with units of min^−1^; E_a_ signifies the activation energy in kilojoules per mole (kJ/mol); R is the molar gas constant, and its value is R = 8.314 J/(mol·K); n indicates the reaction order; m_0_ represents the original mass of the coal in grams (g); and m_b_ is the mass of the coal at time "b" in grams (g).

The relationship between the change in coal mass and the release of heat energy can be expressed as:

8 $${\text{dm}}_{\text{b}}\text{=}\frac{\text{-dQ}}{\text{q}},$$

where Q represents the actual heat release of coal in joules (J); q is the heat of reaction per unit mass of coal (J/g). During the low-temperature oxidation stage, the change in coal mass is negligible, meaning that m_b_ = m_0_. By substituting Eqs. (2) and (3) into Eq. (1) and taking the natural logarithm of the equation, we can obtain Eq. (4).

9 $${\text{ln}}\left( {\frac{{{\text{dQ}}}}{{{\text{dT}}}}\frac{\beta }{{{\text{q}}m_{0} }}} \right){ = } - \frac{{\text{E}}}{{\text{R}}}\frac{{1}}{{\text{T}}}{\text{ + lnA}}{.}$$

## Results and discussion

### Analysis of the effect of pre-oxidation time on the oxidation characteristics of coal oxygen reaction

#### Characteristic temperature analysis

**Figure 3:**
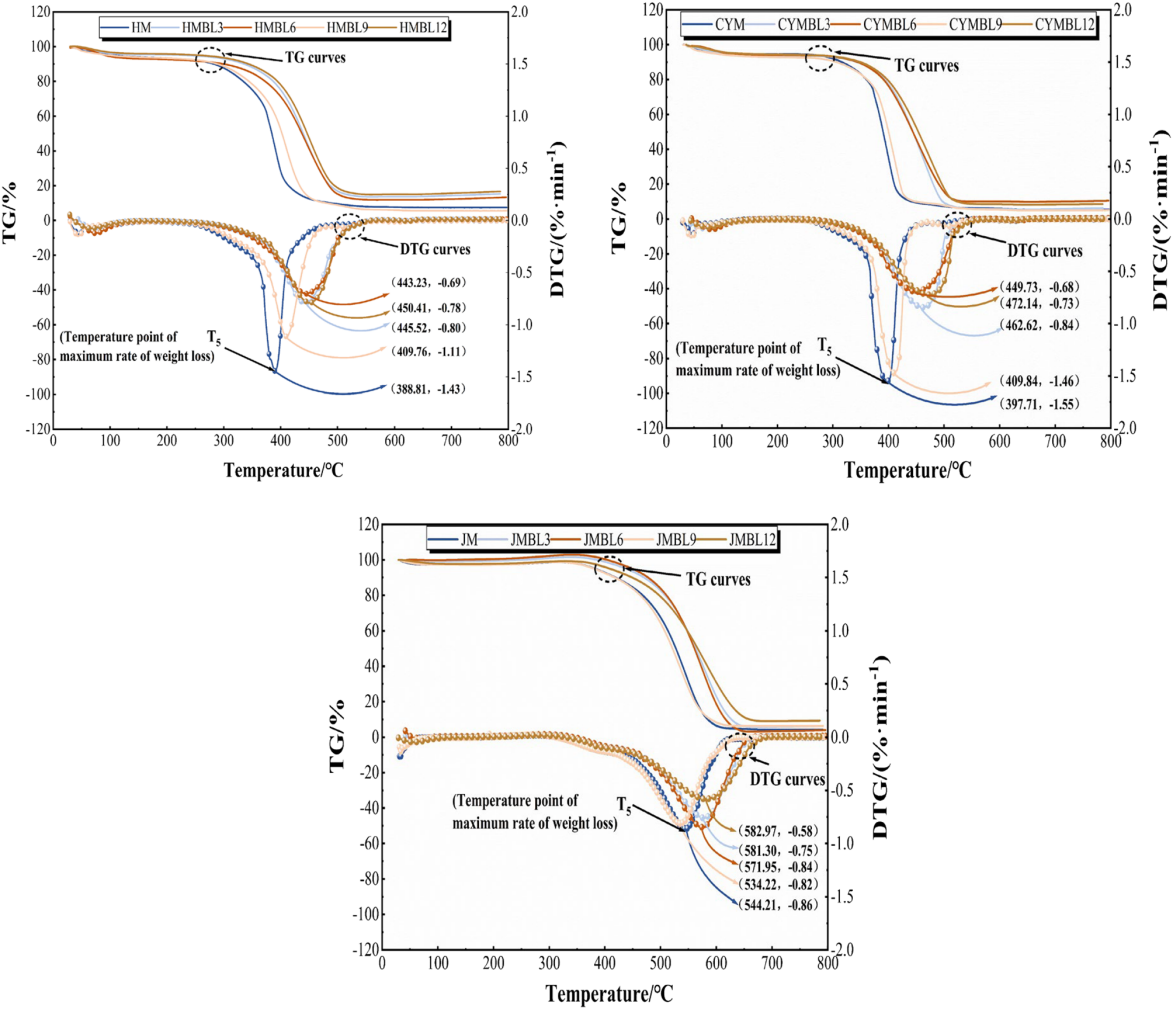
TG-DTG curves of raw and pre-oxidized coal.

To analyze the effect of BL time on coal spontaneous combustion characteristics, simultaneous thermal analysis experiments were conducted on three types of coals with different degrees of metamorphism. The experimental results are presented in Fig. 3.

To analyze the heat-losing properties of the pre-oxidized coal in detail. The characteristic temperatures T_1_, T_4_, T_6_, and the mass change rates of stages 1, 2, and 3, which changed a lot in the heat weight loss process, were analyzed and compared, as displayed in Table 2.

**Table 2:** Characteristic parameters of raw and pre-oxidized coal.

Coal sample	Characteristic parameters					
	T_1_ (℃)	T_4_(℃)	T_6_(℃)	Rate of change in quality (%)		
				Stage 1	Stage 2	Stage 3
HM	132.5	360.7	549.8	− 5.21	− 0.50	− 66.12
HMBL3	140.5	352.8	540.3	− 6.47	− 0.74	− 62.65
HMBL6	147.1	348.0	533.2	− 6.96	− 0.84	− 59.01
HMBL9	153.1	346.1	528.2	− 7.06	− 0.90	− 57.70
HMBL12	136.1	379.2	551.2	− 3.05	− 0.47	− 56.50
CYM	127.5	379.1	550.9	− 5.01	− 0.43	− 70.30
CYMBL3	136.3	369.2	542.1	− 6.27	− 0.64	− 66.15
CYMBL6	143.3	365.9	536.2	− 6.49	− 0.73	− 63.55
CYMBL9	146.9	363.4	532.2	− 6.8	− 0.81	− 61.28
CYMBL12	142.5	394.1	556.5	− 4.46	− 0.40	− 59.64
JM	70.8	472.5	678.1	− 1.82	1.47	− 74.41
JMBL3	106.1	466.1	672.1	− 2.62	2.09	− 70.72
JMBL6	111.9	461.8	667.8	− 2.91	2.37	− 67.01
JMBL9	113.5	457.1	663.3	− 3.12	2.49	− 65.53
JMBL12	108.6	478.7	677.0	− 1.33	1.41	− 64.64

Based on the observations from Fig. 3, it is evident that the thermal analysis curves of the different types of coals with different degrees of metamorphism show significant changes with increasing BL time, with BL9 coal exhibiting the most pronounced effect. To further analyze the influence of BL time on coal spontaneous combustion, the characteristic temperatures of the reaction process will be analyzed.

Based on the characteristic temperatures of the coal under different pre-oxidation times indicated in Table 2, the following conclusions can be drawn: for BL3, BL6, and BL9 coals, their T_1_ values gradually increase, while the T_1_ value of the BL12 coal decreases. This is because volatiles are released from the coal earlier than from the raw coal before BL9, which results in a degradation of the quality of the coal samples. By the BL12 stage, the pore structure in the coal sample increases, promoting its combination with oxygen and enhancing its physical adsorption capacity^32^.

On the other hand, the T_4_ values for HM, CYM, and JM decreased from 360.7 °C, 379.1 °C, and 472.5 °C in the raw coal up to 346.1 °C, 363.4 °C, and 457.1 °C in BL9, then increased to 379.2 °C, 394.1 °C, and 478.7 °C in BL12. This suggests that, in comparison to the raw coal, before BL9, the pre-oxidized coal requires lower external temperatures and energy for oxidation reactions and is, therefore, more susceptible to autoignition. However, by the BL12 stage, the reactive functional groups were overconsumed, resulting in a slower rate of chemisorption of oxygen. On the whole, the T_6_ values of BL3, BL6, and BL9 coal samples are smaller than those of raw coal, indicating that the oxidation rate is faster in these three time periods.

#### Analysis of weight loss characteristics at different stages

**Figure 4:**
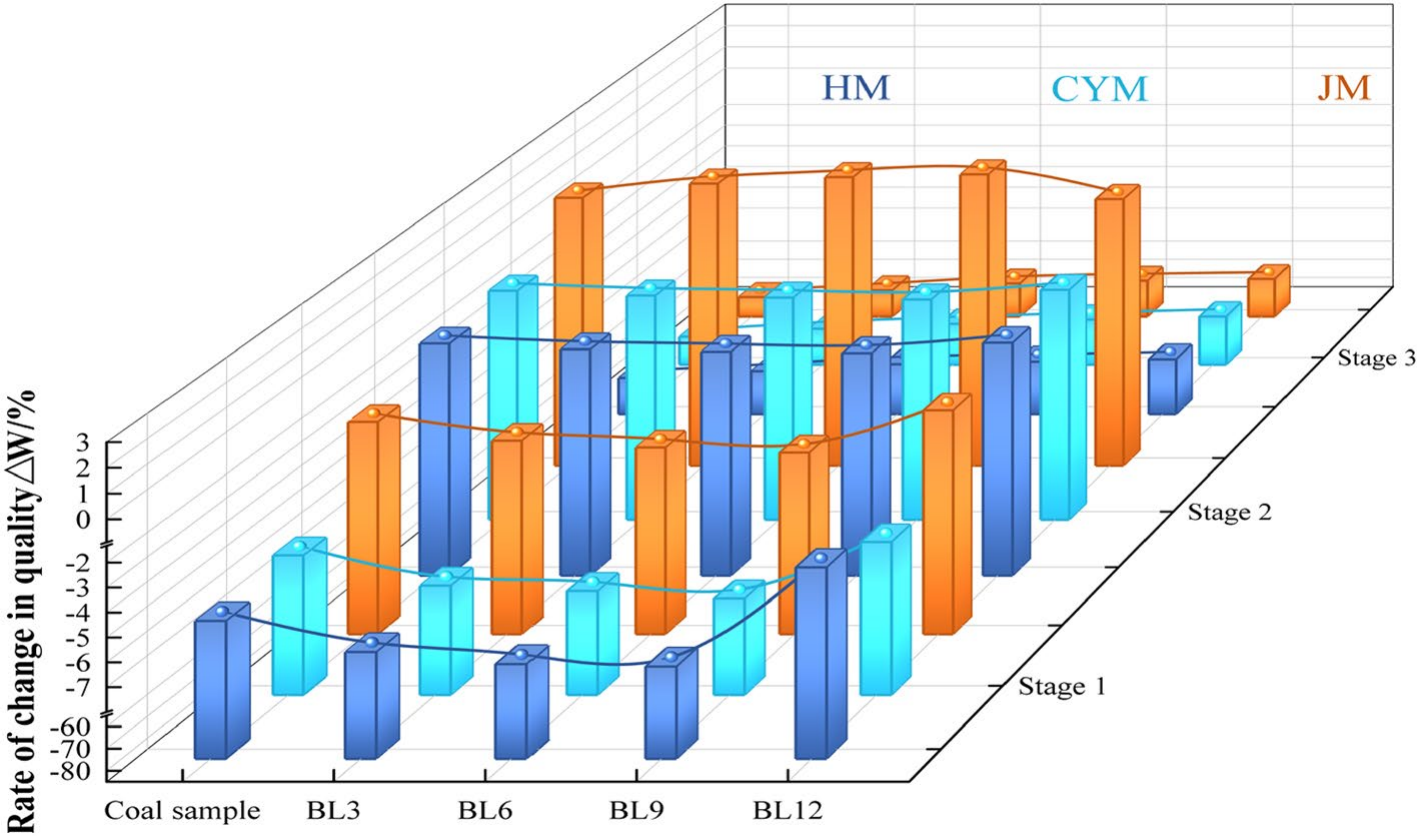
Rate of change in quality of raw and pre-oxidized coal by stage.

Based on the data provided in Table 2, the changing law of coal quality in the stages of primary water evaporation and adsorption, slow reaction, and combustion under various pre-oxidation times can be further investigated. The findings are shown in Fig. 4.

According to Fig. 4, in Stage 1, coal under different pre-oxidation times exhibits a decrease in mass. With the increase in BL time, the mass loss rate of BL3, BL6, and BL9 coals gradually increases, while that of BL12 coal decreases. The mass loss rate of HM, CYM, and JM coals increases from 5.21%, 5.01%, and 1.82% in raw coal to 7.06%, 6.8%, and 3.12% in BL9, and then decreases to 3.05%, 4.46%, and 1.33% in BL12. This is because, before BL9, coal exhibited stronger gas desorption, leading to an increase in the mass loss rate. However, after BL12, the physical adsorption of gases becomes relatively stronger, resulting in a decrease in the mass loss rate. However, after BL12, the physical adsorption of gases becomes relatively dominant.

In Stage 2, HM and CYM coal samples exhibit a mass loss process, while the JM coal sample shows a mass gain process. This is because, for both HM and CYM, there is a higher reduction in the release of gaseous products compared to the adaptation to oxygen from the coal-oxygen composite reaction, which leads to a reduction in quality. On the other hand, for JM, the gas adsorption rate is higher in this stage, increasing mass.

In Stage 3, the mass loss percentage of pre-oxidized coals was less than that of the original coals. With the growth of BL time, there is a gradual decrease in the mass loss rate of BL3, BL6, BL9, and BL12 coals. The percentage of quality loss for HM, CYM, and JM decreased from 74.41%, 70.31%, and 66.12% in raw coal to 64.64%, 59.64%, and 56.50% in BL12. This indicates that pre-oxidized coal starts reacting earlier during the low-temperature oxidation process compared to raw coal, leading to a relatively lower mass loss rate during secondary oxidation^33,34^.

#### Stage response time analysis

**Figure 5:**
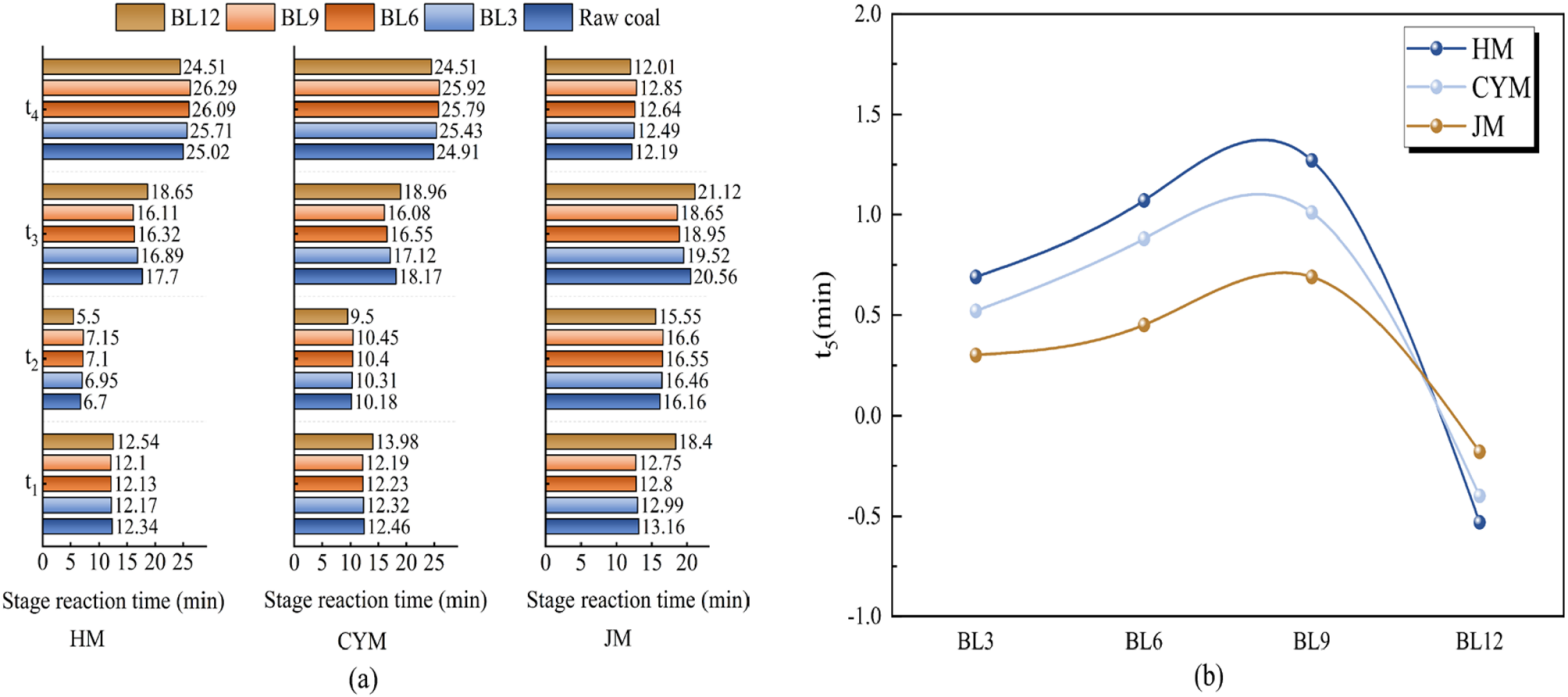
Reaction times for each stage of raw coal and pre-oxidized coal.

To visually represent the coal-oxygen co-combustion rates for different BL times, the reaction times for each stage will be presented in Fig. 5. The durations of oxidation reactions in stages 1 to 4 will be denoted as t_1_, t_2_, t_3_, and t_4_, respectively, while the difference in t_4_ between pre-oxidized coal and raw coal will be represented as t_5_.

At the heat-loss stage of combustion (Stage 4), the coal-oxygen cofiring reaction is essentially over, so the reaction times for Stages 1 through 3 represent the duration of the coal-oxygen cofiring processes. The heat loss experiments of coal at various pre-oxidation times use identical heating rates and temperature zones to ensure that the total time for the entire heat loss reaction is the same. Therefore, the time required for the coal sample to reach complete combustion (t_4_) can reflect the rate of the oxidizing process of coal.

From Fig. 5a, it can be seen that the t_4_ values of BL3, BL6, and BL9 coals gradually increased, while the t_4_ values of BL12 coal gradually decreased. The t_4_ values for HM, CYM, and JM, which were initially 25.02, 24.91, and 12.19 min for raw coal, respectively, increase to 25.99, 26.08, and 14.62 min for BL9 coal, and then decrease to 24.88, 24.35, and 11.55 min for BL12 coal. As illustrated by Fig. 5b, the trends of t_5_ values of BL3, BL6, BL9, and BL12 coals are consistent with the trends of t_4_ values. The t_5_ values for HM, CYM, and JM, which were 0.69, 0.52, and 0.3 min for BL3 coal, respectively, increase to 1.27, 1.01, and 0.69 min for BL9 coal, and then decrease to − 0.53, − 0.4, and − 0.18 min for BL12 coal. These findings show that BL9 pre-oxidized coal reacts faster than raw coal in oxidation, while BL12 coal reacts slower than raw coal in oxidation. This suggests that BL9 pre-oxidized coal produces a large number of peroxides when exposed to air for a long period, and therefore the coal-oxygen co-combustion reaction is faster^35–37^. On the other hand, the slower coal-oxygen co-combustion reaction of BL12 coal is due to the excessive consumption of certain reactive functional groups in the coal, which results in a lower rate of oxidative chemistry adsorption.

#### Combustion characteristics analysis

To quantitatively analyze the thermal weight loss characteristics of coals with different BL times, we employed the combustion characteristic parameter calculation method described in Sect. 2.3.2. Subsequently, we conducted a comparative analysis of these parameters and presented the quantitative analysis results of the characteristic parameters in Table 3.

**Table 3:** Combustion characteristics parameters of raw coal and pre-oxidized coal.

Coal sample	Combustion characteristic parameters				
	(dW_max_)	D	Comprehensive combustion performance parameters		
			C_b_	S	H_f_
HM	− 1.43	2.73	1.08	0.11	3.43
HMBL3	− 0.80	2.21	1.42	0.05	4.54
HMBL6	− 0.78	2.01	1.81	0.03	5.11
HMBL9	− 1.11	1.93	1.98	0.02	5.42
HMBL12	− 0.49	1.71	0.91	0.01	3.33
CYM	− 1.55	2.42	0.64	0.15	3.11
CYMBL3	− 0.84	2.01	0.91	0.10	4.1
CYMBL6	− 0.73	1.85	1.01	0.06	4.43
CYMBL9	− 1.46	1.63	1.06	0.04	4.51
CYMBL12	− 0.68	1.42	0.53	0.03	2.89
JM	− 0.86	1.78	0.39	0.21	2.98
JMBL3	− 0.75	1.41	0.47	0.14	3.65
JMBL6	− 0.84	1.26	0.53	0.1	3.81
JMBL9	− 0.82	1.05	0.56	0.08	3.93
JMBL12	− 0.58	0.98	0.3	0.06	2.72

From the results in Table 3, it can be seen that the dWmax and D figures of the pre-oxidized coal are smaller than those of the original coal. Additionally, the dW_max_, as well as D values for BL3, BL6, BL9, and BL12 coals, continuously decrease. HM, CYM, and JM show a reduction of 0.94, 0.87, and 0.28 wt % min^−1^, respectively, in dWmax, and a decrease of 1.02, 1.00, and 0.80 wt % min^−1^ ℃^−2^, respectively, in D. These findings suggest that the burning and combustion performance of these coals is weaker than original coal in the later stages of burning. This is largely due to the effect of ash content, which not only impedes heat transfer within the coal body but also makes it more difficult to burn the coal completely. In addition, since S represents the overall combustion characteristics of the coal, the outcomes are supposed to be the same as the results of D.

Regarding the H_f_ values for BL3, BL6, BL9, and BL12 coals, they gradually increase until 9 months and then decrease at 12 months. HM, CYM, and JM show an increase in H_f_ from 3.43, 3.11, and 2.98 wt %^−2^ min^2^ ℃^2^ for raw coal to 4.96, 4.92, and 4.41 wt %^−2^ min^2^ ℃^2^ for BL9 coal, and then decrease to 3.21, 3.01, and 2.72 wt %^−2^ min^2^ ℃^2^ for BL12 coal. Indeed, the observation that HM, CYM, and JM exhibit higher combustion rates and short-term burning intensities than raw coal before BL9, resulting in a shorter reaction duration, is consistent with the findings presented in the preceding section. This phenomenon can be attributed to the pre-oxidation sequence, which mobilizes several active functional groups in coals, allowing them to enter the co-combustion process more quickly and increasing the intensity of the reaction at a given time.

However, noteworthy is that the pre-oxidation procedure also results in the loss of some of the functional groups in the coal, which ultimately leads to poorer combustion characteristics (S) of pre-oxidized coals compared to raw coals. Despite the increase in initial combustion rate, the overall combustion performance of the pre-oxidized coal was negatively affected because of the loss of these functional active sites during the pre-oxidation treatment.

### Thermokinetic analysis of the effect of pre-oxidation time on the coal-oxygen reaction process

**Figure 6:**
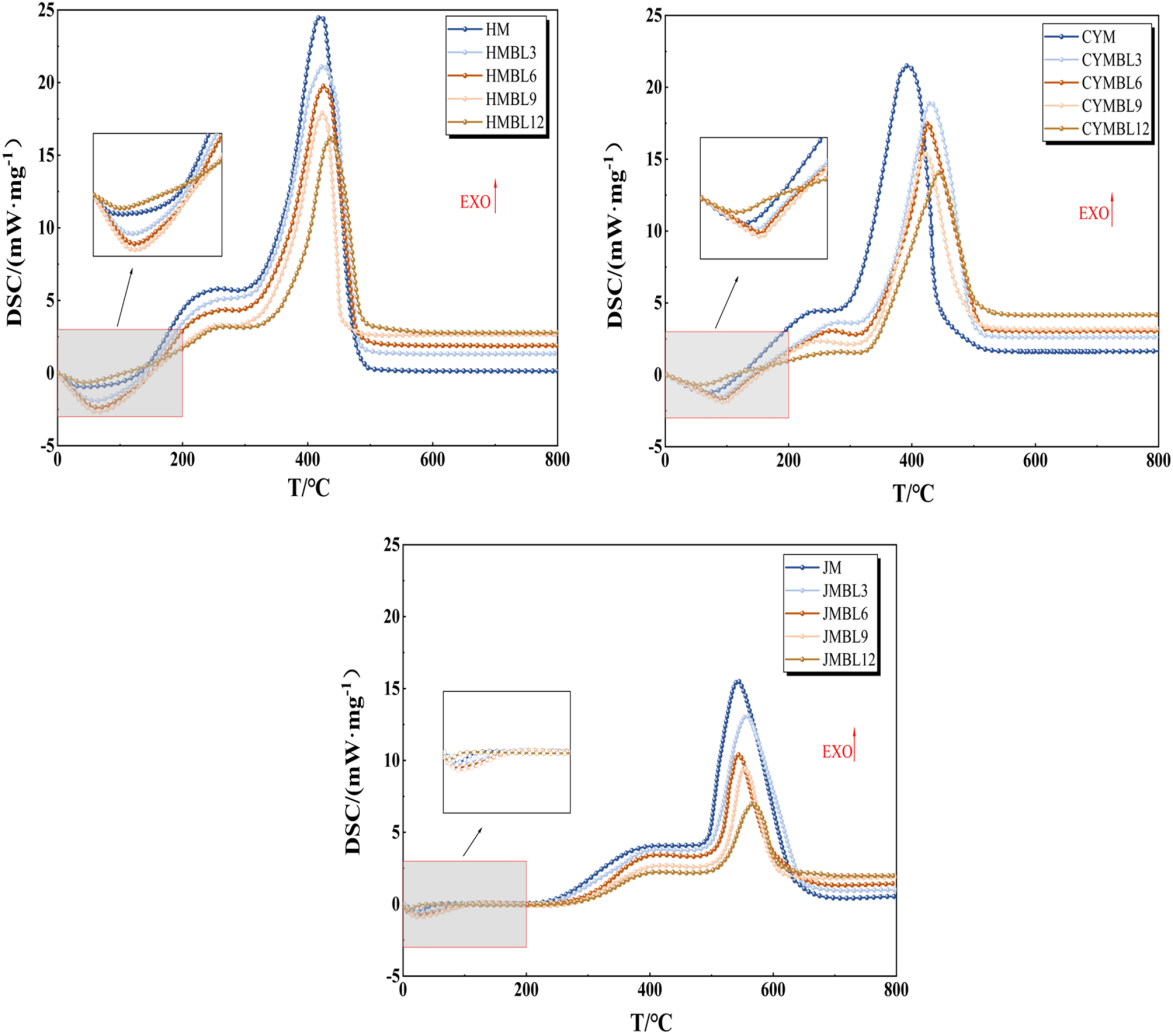
DSC curves of raw coal and pre-oxidized coal.

In simultaneous thermal analysis experiments, the heat released by coal oxidation reactions can be assessed by analyzing changes in heat fluxes. The results of the test are shown in Fig. 6.

Observing Fig. 6, it can be found that the heat flow profiles of the pre-oxidized and raw coals have similar trends to the temperature changes. However, there are significant differences in the thermal flow rate variations among coal samples with different BL times. With increasing BL time, pre-oxidized coal requires noticeably more time for endothermic reactions compared to raw coal, and its overall thermal flow curve lags behind that of raw coal. Overall, the most pronounced changes in the thermal flow curve are observed for BL9 coal, evident in both the time required for endothermic reactions and the values of the thermal flow rate. Conversely, the thermal flow curve of BL12 coal, on the whole, exhibits an advance compared to raw coal. This suggests that different BL times have a considerable impact on the native moisture, gases, and active structures within the coal, leading to modifications in its low-temperature exothermic characteristics.

To calculate the thermodynamic parameters of the accelerated oxidation stage and the rapid oxidation stage for raw coal and pre-oxidized coal during low-temperature oxidation, values of ${\text{ln}}\left(\frac{\text{dQ}}{{\text{dt}}}\frac{\beta }{{\text{q}}{m}_{0}}\right)$ and T^−1^ were computed at intervals of 5 °C, and these calculated values were subjected to fitting. The results are summarized in Table 4.

**Table 4:** Kinetic parameters of raw and pre-oxidized coal.

Coal sample	Stage	E_a_/(kJ·mol^−1^)	lnA/min^−1^	R^2^
HM	1	26.46	4.5	0.9981
	2	54.51	0.38	0.9959
HMBL3	1	35.63	3.29	0.9792
	2	35.21	4.45	0.9736
HMBL6	1	40.32	2.45	0.9875
	2	25.67	5.67	0.9921
HMBL9	1	45.38	3.97	0.9678
	2	15.95	6.39	0.9546
HMBL12	1	19.01	1.81	0.9967
	2	59.51	3.36	0.9982
CYM	1	32.51	0.73	0.9548
	2	65.82	0.63	0.9846
CYMBL3	1	55.39	3.61	0.9478
	2	45.15	2.68	0.9945
CYMBL6	1	70.42	7.94	0.9975
	2	25.15	3.45	0.9946
CYMBL9	1	75.47	11.15	0.9546
	2	20.77	4.05	0.9846
CYMBL12	1	28.49	2.36	0.9987
	2	70.81	1.44	0.9975
JM	1	45.61	3.93	0.9949
	2	73.09	3.39	0.9915
JMBL3	1	65.29	2.56	0.9968
	2	50.15	2.71	0.9478
JMBL6	1	70.27	1.65	0.9572
	2	30.31	3.98	0.9846
JMBL9	1	80.49	0.88	0.9873
	2	28.12	4.23	0.9915
JMBL12	1	35.31	5.25	0.9968
	2	73.82	6.8	0.9478

**Figure 7:**
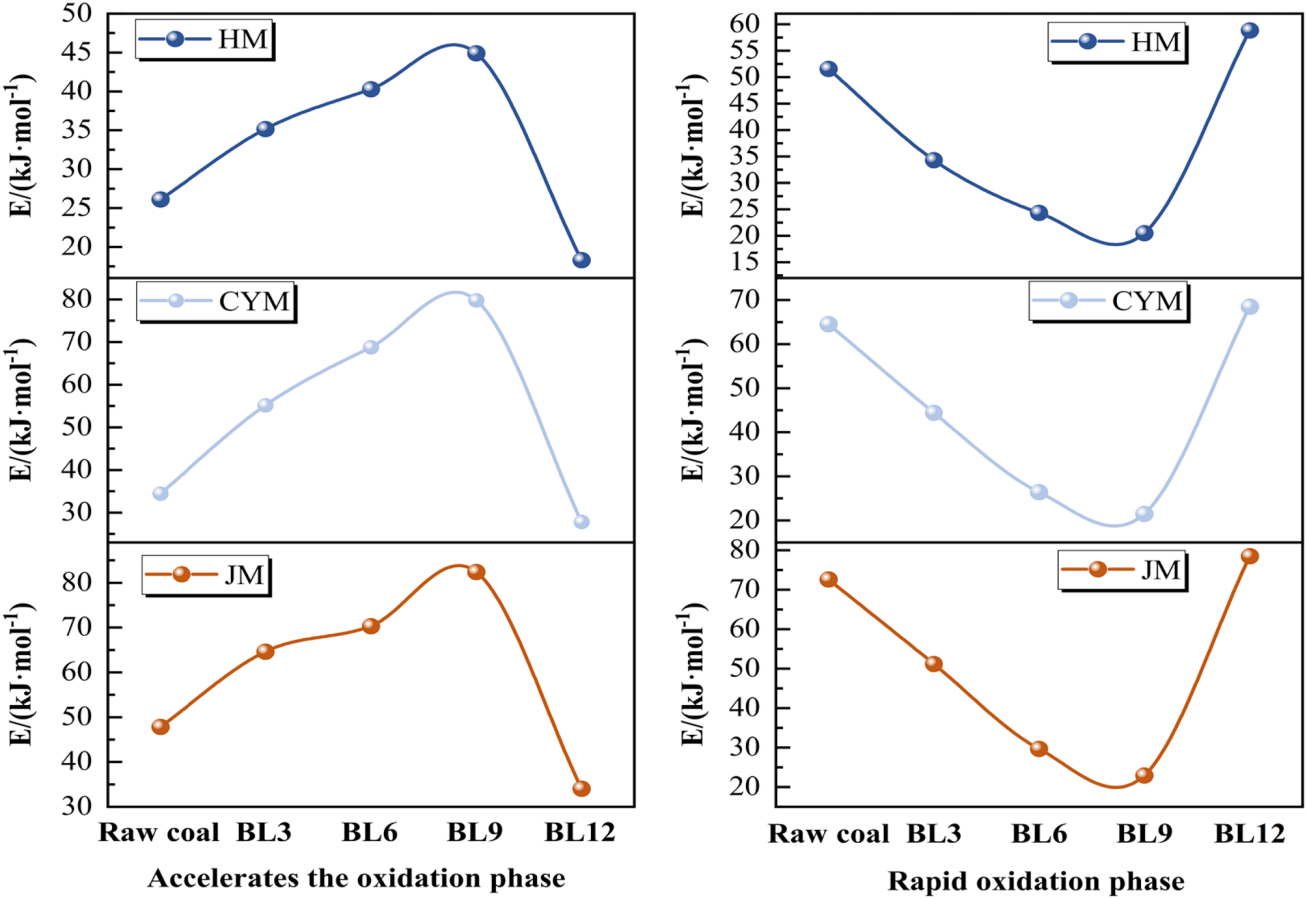
Stage activation energy change curves for raw and pre-oxidized coals.

To more visually express the coal-oxygen complex reaction rates of coals with different pre-oxidation times, the activation energy changes of each stage of accelerated and rapid oxidation are shown in Fig. 7.

Based on the activation energy data, it was observed that the activation energies of BL3, BL6, and BL9 coals gradually increased during the accelerated oxidation stage, while the activation energies of BL12 coal gradually decreased. HM, CYM, and JM exhibit an increase in activation energy from 26.46, 32.51, and 45.61 kJ·mol^−1^ for raw coal to 45.38, 75.47, and 80.49 kJ·mol^−1^ for BL9 coal, and then decrease to 19.01, 28.49, and 35.31 kJ·mol^−1^ for BL12 coal. This is because, before BL9, the active functional groups in the pre-oxidized coal have already undergone partial consumption. This results in the subsequent reactions requiring higher temperatures or more energy to sustain, significantly increasing the difficulty of the coal-oxygen composite reaction. On the other hand, BL12 coal exhibits a higher porosity compared to the original coal, making it more prone to undergoing reactions.

Conversely, the trend of activation energy for the rapid oxidation stage in BL3, BL6, BL9, and BL12 coals is opposite to that of the acceleration oxidation stage. HM, CYM, and JM show a decrease in activation energy from 54.51, 65.82, and 73.09 kJ·mol^−1^ for raw coal to 15.95, 20.77, and 28.12 kJ·mol^−1^ for BL9 coal, and then increase to 59.51, 70.81, and 80.82 kJ·mol^−1^ for BL12 coal. It is since the energy required for the transition of the stable aromatic ring structure and other macromolecular structures in the pre-oxidized coal from the ordinary state to the activated state is partially released when the coal-oxygen co-combustion reaction is carried out at higher temperatures during the pre-oxidation procedure. Thus, when the pre-oxidized coal enters the rapid oxidation phase, the energy required for the activation of the aromatic ring structure and other macromolecular structures is reduced and reactions are more likely to occur. However, in BL12 coal, after prolonged oxidation, the reactive sites in the coal samples are overconsumed and the reaction occurs with greater difficulty compared to the original coal.

### Analysis of the effect of different pre-oxidation times on the reactivity of chemical structures in coal

According to the research conducted by Ibarra et al.^38–40^, the peaks are broadly categorized into four regions. Among these, the intervals from 1000 to 1800 cm^−1^, 2800 to 3000 cm^−1^, and 3000 to 3600 cm^−1^ represent the primary distribution regions of active functional groups in coal. Thus, our analysis is mainly focused on examining and understanding these three specific intervals.

**Figure 8:**
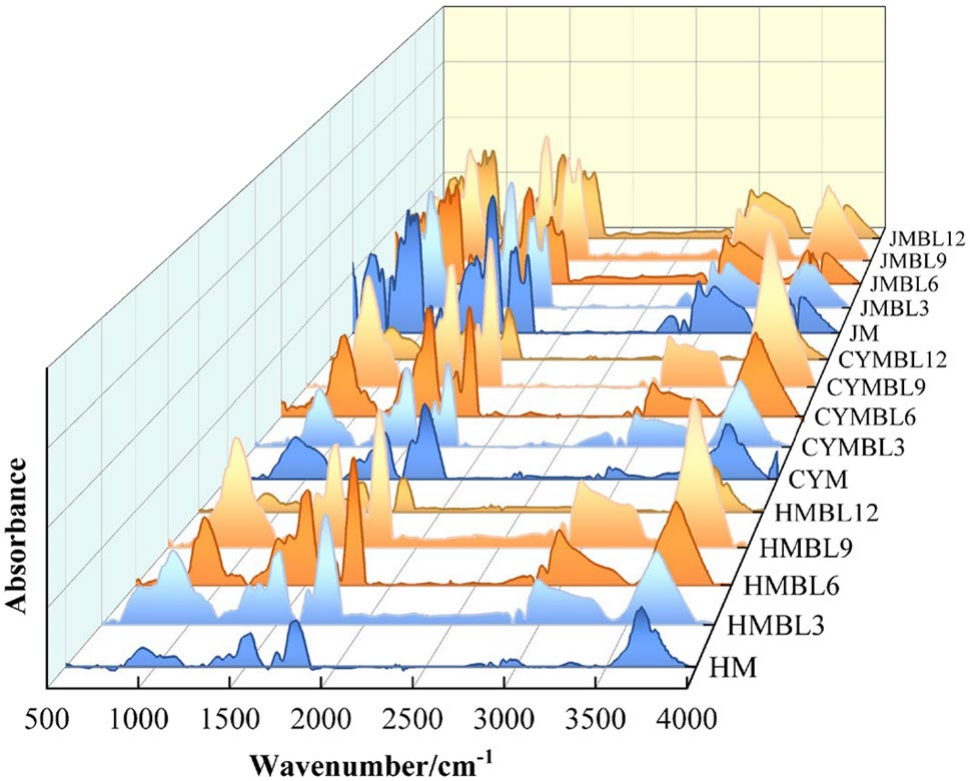
Infrared spectra of raw and pre-oxidized coal.

**Figure 9:**
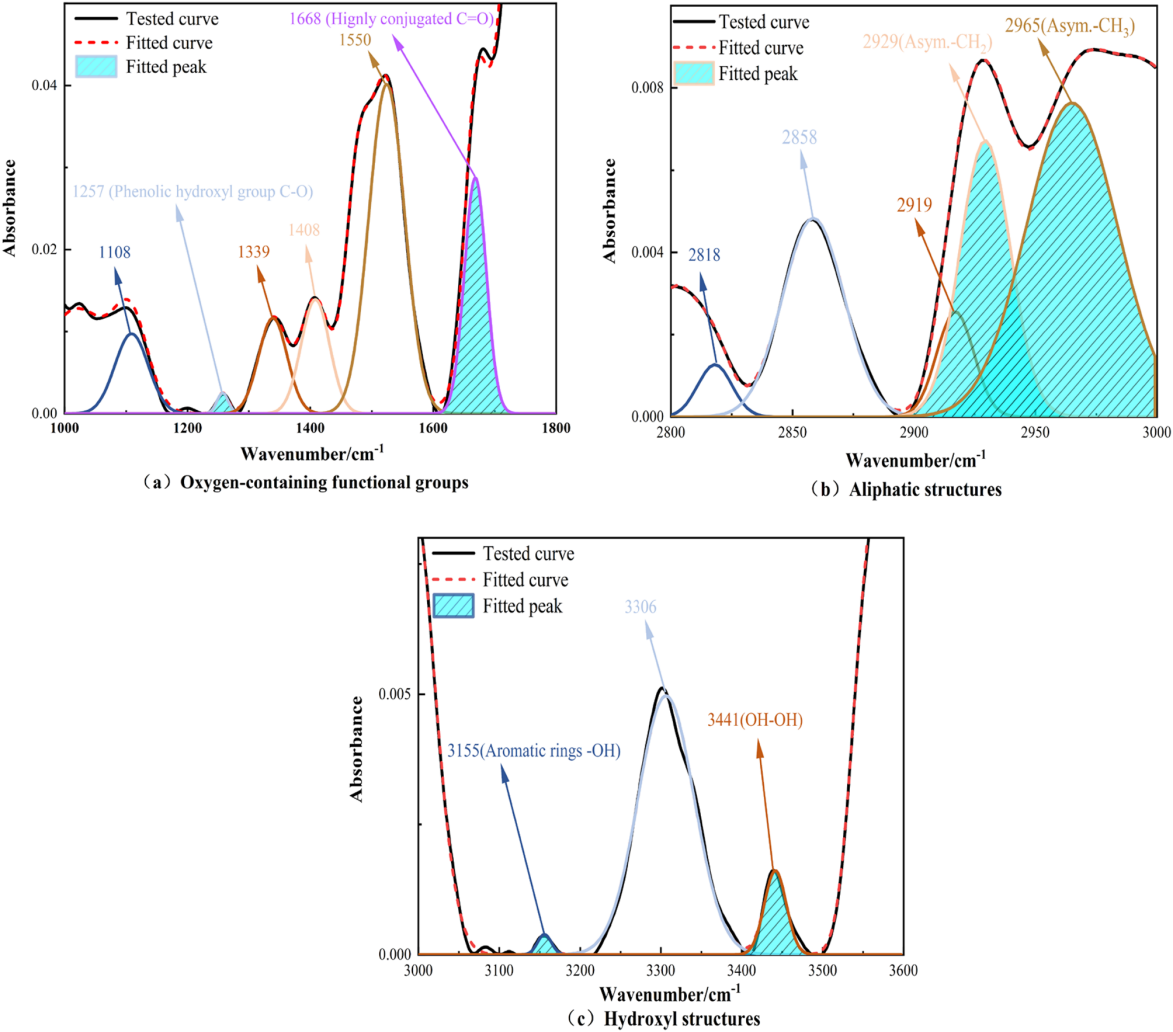
Oxygen-containing functional groups, aliphatic structure, and hydrogen bond peak fitting results.

From the findings in Fig. 8, the absorption peaks corresponding to the active structures in coal changed significantly at different BL times. To further quantitatively investigate the changes in the content of reactive structures in coal during low-temperature temperatures, peak fitting analysis on the experimental data^41^. The concrete findings are presented in Fig. 9.

**Figure 10:**
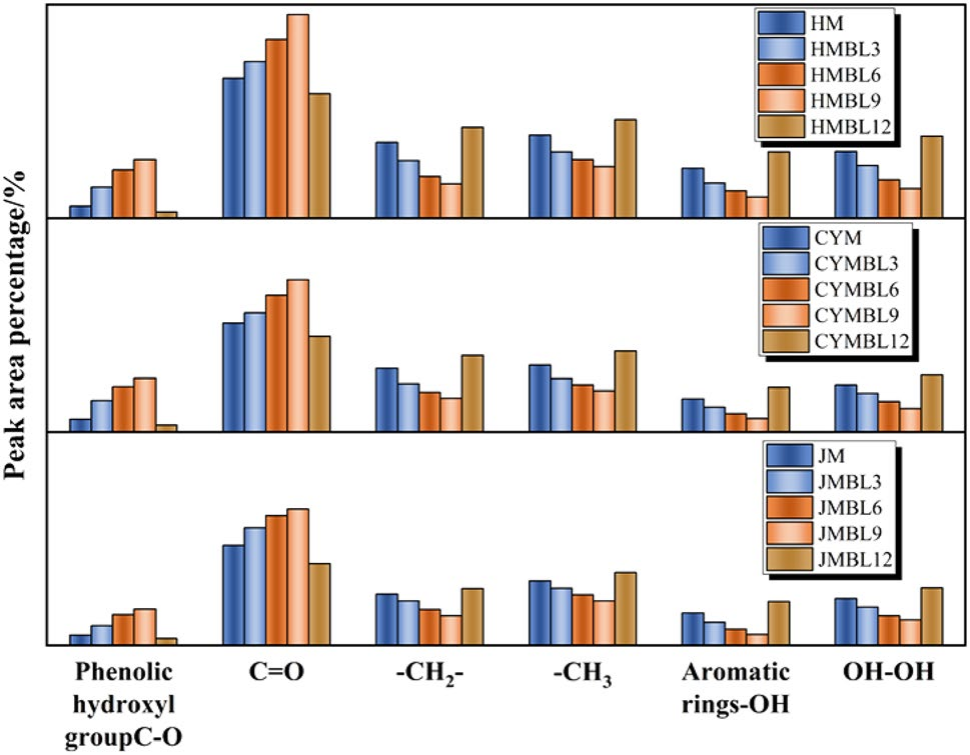
Comparison of the Relative Content of Functional Groups in Raw Coal and Pre-Oxidized Coal.

Based on the experimental results, we observed that the reflection peaks of some active functions in coal changed significantly with the BL time. Specifically, the detection peak around 1257 cm^−1^ is related to the phenolic hydroxyl group (C–O), the detection peak around 1668 cm^−1^ is related to carbonyl group (C = O), the detection peak around 2929 cm^−1^ is related with –CH_2_– anti asymmetric stretch, the detection peak around 2965 cm^−1^ is related with –CH_3_ anti asymmetric stretch, the detection peak around 3155 cm^−1^ is related with aromatic -OH, the detection peak around 3441 cm^−1^ is related with OH–OH, the detection peak around 3441 cm^−1^ is related with hydrogen, and the detection peak around 3441 cm^−1^ is related with OH–OH, the detection peak around 3441 cm^−1^ is related with hydrogen. The absorption peaks around 2965 cm^−1^ correspond to –CH_3_ asymmetric stretching, 3155 cm^−1^ with aromatic –OH, and 3441 cm^−1^ with OH-OH hydrogen bonding, and all of these absorption peaks have been significantly changed. Therefore, we focused on analyzing these peaks and presented the results in Fig. 10.

The relative content of functional groups in coal under different BL times is presented in Fig. 10. From the graph, it is evident that the content of C = O exhibits significant fluctuations with increasing BL time. Specifically, the C = O content in BL3, BL6, BL9, and BL12 coals gradually rises to BL9 and then declines by BL12. For HM, CYM, and JM, the C = O content increases from their original values of 34.05%, 33.07%, and 28.11% to 49.53%, 46.22%, and 38.32% at BL9, before decreasing to 30.26%, 29.03%, and 23.06% at BL12. The maximum C = O content reaches 1.45 times that of the original coal. These observations indicate that C = O plays a more prominent role in coal-oxygen complex reactions. The critical functional group influencing BL coal self-combustion is C = O.

Meanwhile, as BL time increases, the trends of aromatic –OH, OH–OH, –CH_3_, and –CH_2_ changes are similar but opposite to those of phenolic hydroxyl (C–O) and carbonyl (C = O). At the apparent level, this is because fatty hydrocarbons and –OH groups are consumed to form some oxygen-containing functional groups during the coal-oxygen co-combustion process. On a microscopic level, as BL time increases, fatty hydrocarbons are attacked and broken by oxidation, resulting in a decline in fatty hydrocarbon content. Consequently, a large number of exposed reactive sites on the coal are reacted from oxygen to form functional groups such as phenolic hydroxyl groups (C–O) and carbonyl groups (C = O)^42–44^. The increase in their content indicates an enhancement in coal activity.

Furthermore, the stability of ring hydrogen bonds and intramolecular hydroxyl hydrogen bonds in coal decreases^45–47^, making them more susceptible to breaking. As a result, the weakening of hydrogen bonding forces in coal molecules reduces the structural stabilization of the coal and facilitates the overall reaction process. However, after BL12, certain of the groups in the coal samples are overconsumed, which leads to a gradual decrease in the reaction rate and inhibition in the overall process of coal decomposition.

Long-term pre-oxidation at room temperature induces significant phase-dependent changes in the oxidation characteristics of coal. However, this study did not investigate the sustained variations in coal's oxidation characteristics and microstructure during extended pre-oxidation. Hence, in future research directions, it is essential to explore the enduring influence of room temperature pre-oxidation time on coal. This exploration should encompass the application of new research methods or experimental techniques, along with a comprehensive multi-faceted analysis. These methods will aid in extending the current research domain, thereby enhancing the depth and breadth of the research.

## Conclusions

To investigate the impact of BL time on the oxidative reaction characteristics and microstructural changes of coals with varying degrees of metamorphism, comparative STA and FTIR experiments were conducted on brown coal, long-flame coal, and coking coal subjected to different BL times. After rigorous analysis, the following conclusions were drawn:

1. STA experiments were employed to compare and analyze the oxidative reaction characteristics of raw coal and coals subjected to varying BL times (BL3, BL6, BL9, BL12). Thermodynamic calculations revealed that the activation energy during the rapid oxidation stage progressively decreased for BL3, BL6, and BL9 coals, reaching a minimum of 0.29 times that of the original coal, while BL12 coal exhibited relatively higher activation energy. This indicates that pre-oxidation time facilitates coal spontaneous combustion up to BL9, whereas it inhibits it by BL12.
2. The FTIR analysis reveals that the impact of BL time on C = O is particularly pronounced. In the coal samples from BL3, BL6, and BL9, the C = O content gradually increases, whereas in BL12 coal, there is a slight decrease. The highest C = O content reaches 1.45 times that of the original coal. These findings underscore the pivotal role of C = O in coal-oxygen complex reactions and its significance as a key functional group influencing coal self-combustion.
3. These research findings provide valuable insights for predicting the spontaneous combustion risk of oxidized coal. Such knowledge is instrumental in reinforcing safety regulations and risk mitigation measures within the coal industry, ultimately fostering safer and more efficient resource utilization practices.

## Data Availability

The data used to support the findings of this study are available from the corresponding author upon request.
